# The Connection Between Minor H Antigens and Neoantigens and the Missing Link in Their Prediction

**DOI:** 10.3389/fimmu.2020.01162

**Published:** 2020-06-24

**Authors:** Tuna Mutis, Anastasia Xagara, Robbert M. Spaapen

**Affiliations:** ^1^Department of Hematology, Amsterdam UMC, VU Medical Center, Amsterdam, Netherlands; ^2^Department of Immunopathology, Sanquin Research, Amsterdam, Netherlands; ^3^Landsteiner Laboratory, Amsterdam UMC, University of Amsterdam, Amsterdam, Netherlands

**Keywords:** minor histocompatibility antigen, neoantigen, antigen prediction, antigen identification, reverse antigen identification strategy

## Abstract

For hundreds of thousands of years, the human genome has extensively evolved, resulting in genetic variations in almost every gene. Immunological reflections of these genetic variations become clearly visible after an allogeneic stem cell transplantation (allo-SCT) as minor Histocompatibility (H) antigens. Minor H antigens are peptides cleaved from genetically encoded variable protein regions after which they are presented at the cell surface by HLA molecules. After allo-SCT with minor H antigen mismatches between donor and recipient, donor T cells recognize the minor H antigens of the recipient as foreign, evoking strong alloreactive immune responses. Studies in the late eighties have discovered that a subset of minor H antigens are encoded by hematopoietic system-specific genes. After allo-SCT, this subset is strictly expressed on the hematopoietic malignant cells and was therefore the first well-defined highly immunogenic group of tumor-specific antigens. In the last decade, neoantigens derived from genetic mutations in tumors have been identified as another group of immunogenic tumor-specific antigens. Therefore, hematopoietic minor H antigens and neoantigens are therapeutic equivalents. This review will connect our current knowledge about the immune biology and identification of minor H antigens and neoantigens leading to novel conclusions on their prediction.

## Introduction

### Minor H Antigens: From Enigmatic to Well-Defined Transplantation-Antigens

Today, more than six decades after the first application of allogeneic stem cell transplantation (allo-SCT), scientists and clinicians are still impressed by the therapeutic Graft-versus-Tumor (GvT) effect established by donor T cells administered along with stem cells into the recipient (1). This therapeutic effect can be so powerful that patients can remain in long term remissions, even may be cured, after transplantation (2, 3). Therefore, allo-SCT is still being widely applied for several recurrent hematological malignancies, even though the therapeutic effects of allo-SCT are strongly associated with the development of life-threatening Graft-versus-Host-Disease (GvHD). The main mediators of GvHD as well as GvT are the alloreactive donor T cells directed at recipient antigens that are absent in the donor, responding to Major Histocompatibility Complex (MHC; HLA in humans) molecules at the cell surface (4). However, GvT and especially GvHD still occur in about 40% of patients whose stem cell donors are completely HLA-identical, indicating the existence of an additional transplantation antigen system (5). These transplantation antigens were originally designated as minor Histocompatibility (H) antigens (6).

The nature of minor H antigens recognized by donor T cells remained an enigma for more than two decades. In the mid nineties, almost a decade after the identification of MHC-bound peptides as T cell epitopes (7) and the demonstration of structure and the peptide binding groove of MHC class I molecules (8, 9), pioneering studies conducted in mice and humans demonstrated that minor H antigens are polymorphic peptides presented by MHC molecules (10, 11). A subgroup of minor H antigens, the male-specific HY antigens, were derived from “male-specific” proteins encoded by genes located on the Y-chromosome (12). All other non-gender related minor H antigens identified to date are encoded by autosomal genes that have gained allelic polymorphism through evolution over thousands of years [reviewed in Oostvogels et al. (13)]. Although some analyses suggested that minor H antigens are mainly derived from oncological relevant genes (14), this idea was not embraced by all investigators. Any non-synonymous coding variation can give rise to an immunogenic minor H antigen after allo-SCT. Of these variations, single nucleotide polymorphisms (SNPs) leading to single amino acid substitutions are currently the most common for the generation of minor H antigens (10, 15–19). But also base-pair insertions, deletions (indels) or copy number variations (CNVs) contribute to the generation of polymorphic peptides that are recognized as minor H antigens at the cell surface (20).

### The Concept of the Minor H Antigen-Targeted Immunotherapy

As soon as the molecular identity of minor H antigens was unraveled, it became clear why several minor H antigen-specific T cells isolated from transplanted patients lysed only hematopoietic cells, including hematopoietic tumor cells but not the cells derived from other tissues such as fibroblasts or keratinocytes (21). In all those cases the target minor H antigen was encoded by genes, which are solely expressed in the hematopoietic system (22). This discovery underlies the concept of minor H antigen-targeted immunotherapy, which aims at targeting hematopoiesis-specific minor H antigens, which would induce GvT without GvHD after allo-SCT. Also the newly developing minor H antigen negative donor-derived hematopoietic system would remain unharmed (23). The development of this concept fueled the efforts to identify new hematopoiesis-specific minor H antigens. To be broadly therapeutically applicable, such minor H antigens are ideally presented by common HLA-alleles and have a balanced population prevalence in order to get frequent minor H antigen disparities between donor and patient (24). Now, almost 25 years later, the research resulted in the identification of about 10 genuinely hematopoiesis-specific minor H antigens (18, 25–33), some of which have been or are being tested in early phase I/II clinical trials. The approaches used in these trials include treatment of allo-transplanted patients with *ex vivo* generated minor H antigen-specific T cells (34, 35), with T cell receptor (TCR)-gene transferred T cells (NCT03326921, ongoing) or vaccination of allo-transplanted patients with recipient- or donor-derived dendritic cells loaded with minor H antigen peptides (13, 36) or with minor H antigen encoding mRNA (NCT02528682, ongoing) (37). Nevertheless, except the HA-1, UTA2-1, and CD19 minor H antigens (25, 27, 29), all hematopoiesis-specific minor H antigens identified till now are either presented by infrequent HLA-alleles or display an unbalanced population frequency, which makes it highly challenging to enroll sufficient minor H antigen mismatched donor-patient pairs in clinical trials. Due to this issue, all current clinical translation attempts are either progressing very slowly (38) or even terminated due to poor accrual (NCT00943293). Thus, the efficient clinical translation of this highly personalized immunotherapy approach is still largely dependent on the development of solid strategies to identify clinically relevant hematopoiesis-specific minor H antigens. These efforts are relevant not only for the application of minor H antigen-targeted immunotherapy but also for immunotherapy aiming at targeting the so-called neoantigens, because the genetic, immunogenic, and therapeutic properties of hematopoietic minor H antigens and tumor-specific neoantigens display extreme similarities.

### Similarities and Differences Between Hematopoietic Minor H Antigens and Tumor-Specific Neoantigens

Minor H antigens are the immunological reflections of evolutionary established genetic polymorphisms, while neoantigens are immunological reflections of tumor-specific genetic mutations (38, 39). Thus, from a genetic point of view, the only difference between these antigens is that minor H antigens are inherited, while neoantigens are not. For subsequent gene expression, antigen processing and HLA-mediated presentation, minor H antigens and neoantigens follow identical rules. These include that HLA class I (HLA-I) antigens are liberated from the polymorphic or mutated regions of intracellular proteins by (immuno)proteasomes in the cytosol, followed by ER-translocation via transporters associated with antigen processing (TAP) in order to be loaded into HLA-I molecules (40). Next to HLA-I restricted antigens that induce CD8^±^ cytotoxic T cells, HLA class II (HLA-II) restricted antigens that induce CD4^±^ T cells can also play important roles in anti-tumor responses. The proteolytic processing of HLA-II restricted minor H antigens and neoantigens is generally regulated by lysosomal enzymes, followed by HLA-DM assisted loading into the HLA-II peptide binding groove (41).

From the immunological point of view, the existence of minor H antigen- and neoantigen-specific T cells in the naïve T cell repertoire is likely similar. Both antigens are foreign to the immune system and therefore there is no negative selection for the high-affinity T cells reactive with minor H antigens and neoantigens in the thymus (42). Consequently, both antigens can induce very potent T cell immune responses. The basic difference is that minor H antigens are solely immunogenic in a minor H antigen mismatched allo-SCT setting, while neoantigens can be readily immunogenic both in the allogeneic and autologous settings (38, 39).

Finally, from a clinical viewpoint, minor H antigens can be encoded by any polymorphic gene and are thus not tumor-specific antigens *per se*, as opposed to neoantigens. In fact, many minor H antigens are expressed by normal tissues and associate with the occurrence of detrimental GvHD as explained above (43). Nonetheless, this latter distinction does not apply for hematopoietic minor H antigens, which are tumor-specific antigens after an allo-SCT, similar to neoantigens (23, 24). This is because after allo-SCT the originally minor H antigen-positive normal hematopoietic system of the recipient is replaced by the minor H antigen-negative donor hematopoietic system. The only cells expressing the hematopoietic minor H antigens are the residual tumor cells. Therefore, it would not be wrong to state that hematopoietic minor H antigens in an allo-SCT setting are the equivalents of tumor-specific neoantigens. It should be noted that the replacement of residual minor H antigen positive host dendritic cells (DCs) after allo-SCT can take longer periods. These residual host DCs can therefore present endogenous hematopoietic minor H antigens to prime hematopoietic minor H antigen-specific T cells without the need for cross-presentation (44). In the case of neoantigens however, cross-presentation of the target antigen by DCs is an absolute requirement, because tumor cells are generally not able to prime T cells. Furthermore, specific targeting of either of these types of antigen is expected to exclusively generate a powerful anti-tumor effect without inducing direct damage to non-malignant cells.

From the therapeutic point of view, one final common and challenging aspect is the execution of clinical studies. As stated above, many minor H antigen-based clinical studies are facing with poor recruitment issues. Similar poor recruitment for adoptive T cell transfer trials is also expected for neoantigens due to the highly personalized character of most tumor-specific mutations. Since vaccination studies can include several antigens in one study, they are more easily applied (45) as compared to adoptive T cell transfer, but their success is still critically dependent on the development of effective (DC) vaccination strategies that can induce robust and long lasting T cell responses (46).

All these similarities between minor H antigens and neoantigens together show that it is of paramount importance to combine the knowledge of both fields toward the effective identification of both types of antigens and their application in the clinic.

### Most Successful Methods for the Identification of Minor H Antigens and Neoantigens

**Figure 1:**
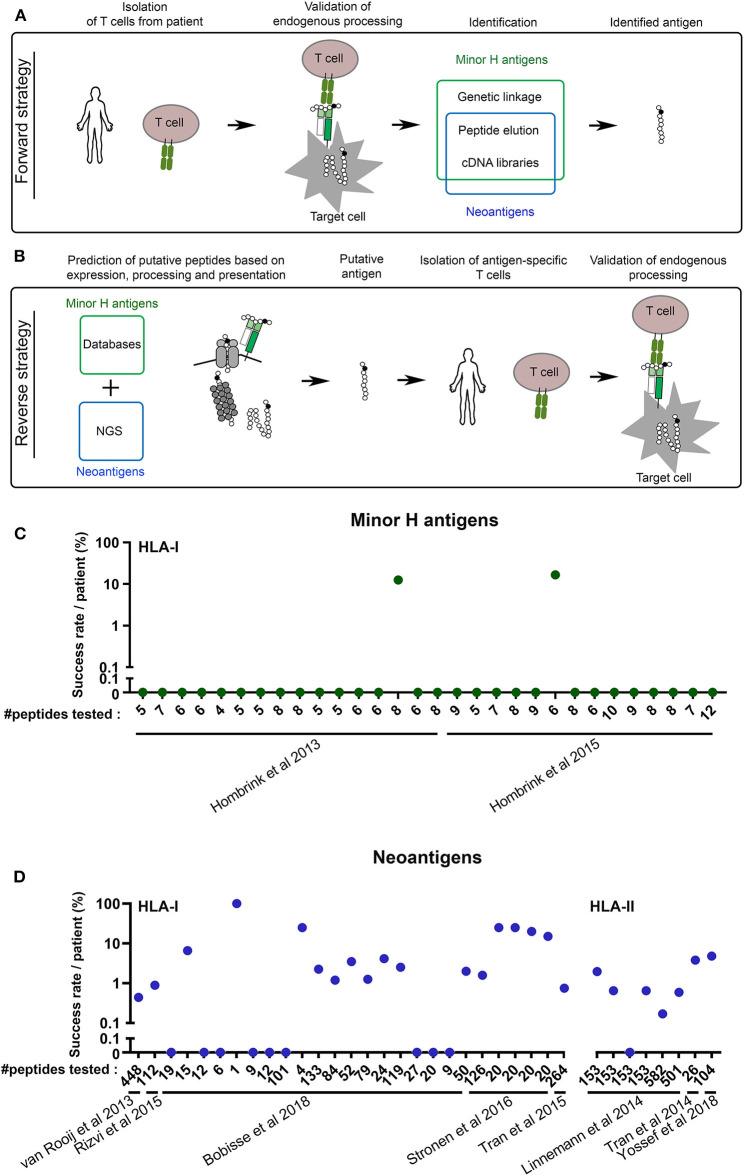
The reverse antigen identification strategy still requires major improvement. **(A)** Schematic overview of the forward antigen identification strategy. **(B)** Schematic overview of the reverse antigen identification strategy. The cartoons depict processing by the proteasome, TAP transporter and HLA class I. NGS, Next Generation Sequencing. **(C,D)** The success rate of the reverse immunology approach is low. **(C)** From two papers in the field of minor H antigens, data were collected about the number of potential minor H antigens that were tested (#peptides tested) vs. the percentage of peptides against which T cell reactivity was detected or raised. Only papers were selected in which reactive T cells were confirmed to recognize the endogenous (or naturally processed) antigen. **(D)** The same was done with eight key papers in the field of neoantigens, five on HLA-I and three on HLA-II antigens.

In general, methods for the identification of a peptide antigen recognized by T cells fall into two main categories. The direct (forward) strategy aims to identify the antigen of a T cell clone that has already been isolated from a patient or a healthy individual. The “reverse” strategy follows the opposite direction through the isolation of a T cell clone that recognizes an *in silico* predicted antigen of interest (Figures 1A,B).

#### Forward Antigen Identification Strategies

There are several forward methods to identify minor H antigens and a fewer to identify neoantigens (Figure 1A). Initially, specialized biochemical peptide elution and fractionation techniques were used to identify peptides recognized by minor H antigen- and tumor-specific T cell clones (10, 12, 18, 25). At the same time, laborious cDNA library screening approaches were utilized to identify minor H antigen and tumor antigen encoding mRNA (47, 48). However, after the discovery that minor H antigens were encoded by inheritable genetic variations, genetic analyses were developed specifically for minor H antigens (32). Over the last 15 years, we and others have advanced these analyses from conventional pairwise linkage analysis into rapid and convenient SNP-based genome-wide association studies (15, 29, 49, 50). Moreover, we have implemented major resolution upgrades of those screens that initially used self-made databases toward publically available databases first from the HapMap Project and later from the 1,000 Genomes Project, which highly improved the success rate of genetic HLA-I and HLA-II restricted minor H antigen identification efforts (49). With these methods, a minor H antigen recognized by a T cell can be identified within 3–4 months (15). It is therefore not surprising that the vast majority of the more than 50 known minor H antigens to date has been identified by genetic linkage analyses (15, 27–29, 32, 33, 49, 51–53). Since neoantigens are not encoded in the germline and thus are not polymorphic in the population, the highly convenient forward genetic approaches are not applicable to their identification.

Despite its evident success, the forward T cell-to-antigen strategy has clear drawbacks when it comes to the identification of minor H antigens with a desired HLA-restriction, population frequency and tissue distribution. Although minor H antigen-specific T cell clones can be readily isolated from many, if not all, allo-transplanted patients, the available T cell isolation techniques cannot be adapted to isolate only those T cell clones with the required characteristics (24). All generated T cell clones need to be tested for the desired HLA restriction and minor H antigen frequency using cell line panels. Moreover, there are no convenient and reliable strategies to select T cell clones directed at minor H antigens expressed only in the hematopoietic system. A better and more convenient control of HLA restriction, population frequency and tissue distribution is key toward more efficient identification of clinically relevant minor H antigens.

#### The Reverse Antigen Identification Strategies

While forward methods hamper at the identification of clinically relevant minor H antigens and neoantigens, opportunities are offered by the “reverse immunology” approach. The reverse method first predicts potential T cell antigens based on *in silico* analyses of polymorphic or mutated genomic sites (Figure 1B). The 1,000 Genomes Project has cataloged most of human polymorphism and is therefore the database of choice for selection of putative minor H antigen encoding variations, preferably with a balanced allele frequency to allow for optimal donor-recipient disparity (54, 55). Because of the personalized character of neoantigens, the current state-of-the-art for mapping individual mutations is through tumor exome sequencing (56). Next tumor transcriptome analyses based on RNAseq or online databases are usually utilized to filter for tumor expression. Candidate minor H antigens should go through an additional selection for selective hematopoietic restricted expression. Finally, algorithms are applied to determine HLA-binding and sometimes the antigen processing efficiency for each possible peptide covering the polymorphism or mutation (56). The combined predictions for HLA-I presented peptides generally provide a score that accounts for the C-terminal cleavage of the protein by the proteasome, the TAP-mediated translocation of the peptide into the ER and the binding of the peptide to HLA with high “on-” and low “off-” rates. The multistep predictions are then validated by isolating the antigen-specific T cells from relevant patients or individuals. The last, but very essential steps are the confirmation that the targeted antigens are naturally processed and that these endogenous antigens are effectively recognized by the isolated T cells or their TCRs (Figure 1B).

The reverse strategy contained several highly challenging aspects until the last decade, which underlie the limited success of minor H antigen or neoantigen identification attempts in that period. Thanks to the recent advances in human genomics [e.g., RNAseq, exome sequencing, 1,000 Genomes Project (54, 56)], tissue expression profiling [e.g., Single Cell Expression Atlas, The Human Protein Atlas, BioGPS (57–59)], antigen processing and binding algorithms [e.g., NetCTL/IEDB (60, 61)] and large-scale peptide-specific T cell detection tools [e.g., UV-exchangeable HLA-I multimers, multimer barcoding (56)], the reverse methodology has majorly improved especially for HLA-I antigens.

This has led to the identification of a vast number of neoantigens, the antigen category for which the forward identification strategies offered only limited options. At the same time, the *in silico* predictions have even led to the generation of multiple libraries of thousands of putative new minor H antigens (55, 62). Nonetheless, these thousands of putative minor H antigens identified by such strategies have only resulted in the actual identification of a handful of minor H antigens (17, 26, 63), because to date none of these reverse strategies account for all minor H antigen-specific features (see below). The success of this gene-to-T cell approach highly depends on the strategy of antigen selection and intensity of T cell isolation efforts. In order to quantify the current efficiency of the reverse strategy, we have analyzed seven recent studies that applied a reverse strategy to identify novel minor H antigens or neoantigens (63–69). We only included studies from which we could extract the number of predicted antigens actually tested, as compared to the number of antigens that were endogenously expressed and against which a true T cell response could be raised. These analyses show that the efficiency of the reverse identification approach is between 0 and 20% (Figures 1C,D). These data argue that the reverse identification pipeline is currently far from optimal, with a dominant pool of false positive predicted candidates against which no T cell reactivity can be detected or raised (Figures 1C,D). One of the causes of suboptimal prediction is the incompleteness of the human reference proteome, despite huge progress in the last two decades. This is illustrated by the fact that many newly identified antigens were derived from supposedly non-coded regions (70–73). Furthermore, different efforts have shown that more stringent selection on expression and HLA binding score improves the success rate (66). Because not all neoantigens or minor H antigens behave according to these stringent criteria, we expect the number of false negative antigens to increase, which could effect the amount of therapeutic opportunities for individual patients in the long term. Thus, there is still much room for improvement of the reverse approach, which in theory is the best directed and straight forward strategy to identify HLA-I restricted hematopoiesis-specific minor H antigens as well as neoantigens.

For HLA-II restricted antigens, the development of a reverse method is even more complicated, because the rules for antigen processing and HLA-binding are more promiscuous and less defined. Nevertheless, some pioneering studies have combined minimalistic *in silico* analyses, without including antigen processing or HLA-binding predictions, but with large plasmid- or peptide-library screening strategies to identify HLA-II restricted neoantigen-specific T cell responses in cancer patients (67, 74, 75). Similar to HLA-I restricted antigens, these studies resulted in a low discovery rate (0–6%, Figure 1D). Recently, predictions for HLA-II binding have been incorporated in these analyses, but endogenous processing of immunogenic peptides was not confirmed (45, 76). So far, no HLA-II restricted minor H antigens have been identified following a reverse strategy. For a more successful identification of HLA-II restricted minor H antigens or neoantigens through reverse strategies, prediction algorithms for peptide processing and HLA-binding, but also cognate T cell detection tools (such as HLA-II multimers) still require revolutionary improvements.

As an additional layer of confirmation before isolating T cells, recent studies applied selection of candidate minor H antigen peptides from a large pool of HLA-I derived peptides as detected by mass spectrometry (Figure 1C) (63, 69, 77). Nonetheless, these studies also generated many false positive candidates, indicating that starting analyses from HLA-derived peptide repertoire may not necessarily compensate the current drawbacks of T cell epitope prediction algorithms.

### Differential Peptide Processing and Presentation Is a Major Opportunity in Minor H Antigen and Neoantigen Reverse Identification

When studying the immunogenicity of genetic variations, it is not sufficient to consider only the antigen processing steps such as peptide cleavage, TAP translocation, HLA binding. The proper execution of these processing steps is definitely required, but not sufficient for the majority of minor H antigens and neoantigens to become immunogenic. This is because the immunogenicity of a polymorphic or mutated peptide depends on the existence of peptide-specific T cells in the (donor) T cell repertoire. The extent of the T cell repertoire against a specific antigen can be negatively affected by the presence of similar antigens in the HLA-presented peptidome during thymic development (78). Since in most cases minor H antigens and neoantigens differ only in a single amino acid from their respective allelic or wildtype counterpart peptides, it is crucial to consider potential effects on the shaping of the T cell repertoire.

If both mutated and wild type peptides are equally well-presented at the cell surface, then T cells may discriminate between these two peptides depending on the position of the amino acid substitution. This is for instance the case for two minor H antigens that both have a single amino acid substitution due to a SNP, HB-1 and ACC1 (Table 1) (26, 32). Separate T cell clones have been isolated that specifically recognize either one or the other allelic peptide at the cell surface (26, 32).

**Table 1:** The majority of minor H antigens (30/50) identified by an unbiased forward strategy are (predicted to be) differentially processed.

**Levels of differential peptide processing**	**Minor H antigen**	**HLA restriction^a^**	**Peptide sequence^b^^,^^c^**	**C-terminal cleavage score^c^^,^^d^**	**TAP score^c^^,^^d^**	**HLA affinity score (nM)^c^^,^^d^**	**References**
Transcription	UGT2B17/A29	HLA-A^*^29:02	**AELLNIPFLY**				(79)
			–				
	UGT2B17/B44	HLA-B^*^44:03	**AELLNIPFLY**				(79)
			–				
	UGT2B17/A2	HLA-A^*^02:06	**CVATMIFMI**				(50)
			–				
	ACC-6	HLA-B^*^44:02/03	**MEIFIEVFSHF**				(31)
			–				
	ZAPHIR	HLA-B^*^07:02	**IPRDSWWVEL**				(80)
			–				
Translation	LRH-1	HLA-B^*^07:02	**TPNQRQNVC**				(28)
			–				
	PANE1	HLA-A^*^03:01	**RVWDLPGVLK**				(81)
			–				
C-terminal or internal proteasome cleavage	ACC-4	HLA-A^*^31:01	ATLPLLCAR	**0.26**	0.68	17	(82)
			ATLPLLCAG	**0.03**	−0.57	11313	
	ACC-5	HLA-A^*^33:03	WATLPLLCAR	**0.26**	0.65	210	(82)
			WATLPLLCAG	**0.03**	−0.59	29173	
	HA-3^e^	HLA-A^*^01:01	VTEPGTAQY	**0.97**	1.25	13	(16)
			VMEPGTAQY	**0.97**	1.31	134	
	SP110^e^	HLA-A^*^03:01	SLPRGTSTPK			54	(83)
			SLPGGTSTPK			155	
	LB-FUCA2-1V	HLA-B^*^0702	RLRQVGSWL	**0.90**	0.48	38	(84)
			RLRQMGSWL	**0.53**	0.48	24	
	LB-GEMIN4-1V	HLA-B^*^07:02	FPALRFVEV	**0.97**	0.04	65	(53)
			FPALRFVEE	**0.24**	−0.78	3208	
	LB-GEMIN4-2V	HLA-B^*^08:01	FPALRFVEV	**0.97**	0.04	23	(71)
			FPALRFVEE	**0.24**	−0.78	405	
TAP transport	HA-8	HLA-A^*^02:01	RTLDKVLEV	0.96	**0.23**	35	(30)
			PTLDKVLEV	0.96	**−0.08**	3665	
HLA-binding	HA-1	HLA-A^*^02:01	VLHDDLLEA	0.95	−0.19	**29**	(25)
			VLRDDLLEA	0.93	−0.18	**321**	
	HA-2	HLA-A^*^02:01	YIGEVLVSV	0.96	0.12	**7**	(10)
			YIGEVLVSM	0.96	0.11	**58**	
	TRIM22	HLA-A^*^02:01	MAVPPCCIGV	0.95	0.17	**620**	(85)
			MAVPPCRIGV	0.89	0.17	**3046**	
	LB-APOBEC3B-1K	HLA-B^*^07:02	KPQYHAEMCF	0.26	0.95	**278**	(53)
			EPQYHAEMCF	0.26	0.82	**9507**	
	LB-BCAT2-1R	HLA-B^*^07:02	QPRRALLFVIL	0.94	0.33	**253**	(53)
			QPTRALLFVIL	0.92	0.30	**2753**	
	DPH1	HLA-B^*^57:01	SVLPEVDVW	0.45	0.50	**217**	(34)
			SLLPEVDVW	0.59	0.44	**1582**	
	LB-TRIP10-1EPC	HLA-B^*^40:01	GEPQDLCTL	0.96	0.26	**176**	(52)
			GGSQDLGTL	0.87	0.21	**15676**	
	LB-C16ORF-1R	HLA-B^*^07:02	RPCPSVGLSFL	0.9	0.38	**643**	(71)
			WPCPSVGLSFL	0.9	0.26	**3010**	
	LB-NCAPD3-1Q	HLA-A^*^02:01	WLQGVVPVV	0.91	0.18	**13**	(71)
			WLRGVVPVV	0.91	0.22	**108**	
	UTA2-1	HLA-A^*^02:01	QLLNSVLTL	0.97	0.46	**39**	(27)
			QLPNSVLTL	0.97	0.45	**222**	
	LB-TMEM8A-1I	HLA-B^*^07:02	RPRSVTIQPLL	0.97	0.41	**11**	(71)
			RPRSVTVQPLL	0.97	0.41	**28826**	
	LB-ERAP1-1R	HLA-B^*^07:02	HPRQEQIALLA	0.96	−0.45	**692**	(53)
			HPPQEQIALLA	0.97	−0.48	**12460**	
	LB-ADIR-1F	HLA-A^*^02:01	SVAPALALSPA	0.89	−0.08	**490**	(86)
			SVAPALALFPA	0.91	−0.08	**1555**	
TCR affinity	HB-1	HLA-B^*^44:03	**EEKRGSLHVW**	0.9	0.29	184	(48)
			**EEKRGSLYVW**	0.89	0.29	188	
	ACC1	HLA-A^*^24:02	**DYLQYVLQI**	0.9	0.20	115	(31)
			**DYLQCVLQI**	0.77	0.20	197	
Yet unknown	LB-ECGF-1	HLA-B^*^07:02	RPHAIRRPLAL	0.91	0.42	9	(73)
			RPRAIRRPLAL	0.91	0.43	5	
	SLC5A1	HLA-B^*^40:02	AEATANGGLAL	0.96	0.49	48	(50)
			AEPTANGGLAL	0.96	0.48	50	
	LB-WNK1-1I	HLA-A^*^02:01	RTLSPEIITV	0.97	0.30	58	(53)
			RTLSPEMITV	0.95	0.30	78	
	LB-NDC80-1P	HLA-A^*^02:01	HLEEQIPKV	0.97	0.09	82	(71)
			HLEEQIAKV	0.97	0.09	184	
	LB-ZDHHC6-1Y	HLA-B^*^07:02	RPRYWILLVKI	0.97	0.23	338	(71)
			RPRHWILLVKI	0.95	0.18	334	
	LB-SON-1R	HLA-B^*^40:01	SETKQRTVL	0.92	0.37	58	(52)
			SETKQCTVL	0.95	0.37	28	
	LB-SWAP70-1Q	HLA-B^*^40:01	MEQLEQLEL	0.94	0.43	178	(52)
			MEQLEELEL	0.92	0.43	141	
	LB-NUP133-1R	HLA-B^*^40:01	SEDLILCRL	0.90	0.28	194	(52)
			SEDLILCQL	0.90	0.28	80	
	P2RX7	HLA-A^*^29:02	WFHHCHPKY	0.95	1.40	6	(34)
			WFHHCRPKY	0.85	1.40	15	
	LB-TTK-1D	HLA-A^*^02:01	RLHDGRVFV	0.89	0.26	30	(51)
			RLHEGRVFV	0.84	0.26	33	
	LB-EBI3-1I	HLA-B^*^07:02	RPRARYYIQV	0.96	0.15	26	(53)
			RPRARYYVQV	0.96	0.15	19	
	LB-ARHGDIB-1R	HLA-B^*^07:02	LPRACWREA	0.42	−0.07	10	(53)
			LPRACWPEA	0.42	−0.07	35	
	LB-SSR1-1S	HLA-A^*^02:01	VLFRGGPRGSLAVA	0.89	−0.14	1403	(87)
			VLFRGGPRGLLAVA	0.86	−0.14	649	
	LB-PRCP-1D	HLA-A^*^02:01	FMWDVAEDL	0.92	0.49	8	(53)
			FMWDVAEEL	0.95	0.49	3	
	LB-MOB3A-1C	HLA-B^*^07:02	CPRPGTWTC	NA^f^	−0.13	442	(71)
			SPRPGTWTC		−0.09	69	
	LB-PNP-1S	HLA-B^*^13:01	TQAQIFDYSEI	0.57	0.28	NA^g^	(71)
			TQAQIFDYGEI	0.4	0.28		
	LB-GSTP1-1V	HLA-B^*^08:01	DLRCKYVSL	0.77	0.24	8	(71)
			DLRCKYISL	0.71	0.24	13	
	C19ORF48	HLA-A^*^02:01	TAWPGAPEV	0.97	0.38	163	(72)
			TAWPGAPGV	0.96	0.38	268	
Predicted not to bind HLA^h^	LB-C19ORF48-2E	HLA-B^*^51:01	TAWPGAPEV	0.97	0.38	24822	(71)
			TAWPGAPGV	0.96	0.38	25920	
	LB-PDCD11-1F	HLA-B^*^07:02	GPDSSKTFLCL	0.97	0.15	7364	(53)
			GPDSSKTLLCL	0.96	0.15	7408	
	LB-ZNFX1-1Q	HLA-B^*^40:01	NEIEDVWQLDL	0.94	0.47	5834	(71)
			NEIEDVWHLDL	0.96	0.47	4026	
	LB-APOBEC3B-1K	HLA-B^*^08:01	KPQYHAEMCF	0.26	0.95	9287	(71)
			EPQYHAEMCF	0.26	0.82	4709	
	LB-CCL4-1T	HLA-A^*^02:01	CADPSETWV	0.15	0.08	8014	(71)
			CADPSESWV	0.29	0.08	9995	

a For B

* 44:02/03 binding mHags, only scores for B*44:03 are depicted.

b *The upper peptide sequence corresponds to minor H antigen and the bottom to allelic counterpart*.

c *Bold values indicate the level of (predicted) differential peptide processing*.

d *Predictions made by NetChop3.1, IEDB and NetMHC4.0*.

e *HA-3 and SP110 are generated through differential internal proteasome cleavage and proteosome-catalyzed peptide splicing*.

f *The C-terminus of the MOB3A antigen is the C-terminus of the MOB3A protein*.

g The HLA-B*13:01 binding prediction is not available at NetMHC4.0, but both peptides harbor the HLA-B*13 binding motif.

h *Because the NetMHC4.0 affinity scores indicated no binding to HLA (>> 1,000 nM), it is currently complicated to assess potential differential HLA-binding, so we excluded these five minor H antigens from the analyses*.

However, if the amino acid alteration is not at a position in the peptide that is exposed to the TCR, and if it is not affecting peptide conformation, then T cells can not discriminate between two of such slightly different peptides. In this case, the allelic or wildtype peptide variants already induce the cognate T cells to be deleted from the T cell repertoire by negative selection in the thymus, similar to self-peptide-reactive T cells (42). Consequently, individuals (donors or patients) lack the majority of the minor H antigen or neoantigen-specific T cells before even being exposed to these antigens. Furthermore, the few T cells in the repertoire that are antigen-specific will have a low affinity TCR with cross-reactivity against the allelic or wildtype peptide. Therapeutic use of such T cells may put patients at risk of detrimental self-recognition on healthy cells, similar to what has been observed for tumor antigens MART-1 and MAGE-A3 (88–91).

Such restrictions do not apply for peptide pairs that are differentially expressed on the cell surface. In fact, for several minor H antigens this is the case. For instance HA-1-, HA-2- and HA-3-specific T cells make no distinction between the allelic peptides (16–18, 25). The strong immunogenicity of such minor H antigens occurs due to the fact that the non-immunogenic peptide has impaired HLA-binding (HA-1) (92), proteasome cleavage (HA-3) (16), TAP translocation (HA-8) (30) or even a yet unknown processing event (HA-2) (Table 1). Similar one-sided lack of peptide presentation occurs if the genetic variation causes loss of conventional gene transcription such as through alternative splicing (31) or loss of genomic DNA (Table 1) (79). Finally, alternative translation events may also cause differential surface expression, for example due to a frame shift (28) or the introduction of a stop-codon (81) (Table 1). Moreover, *in silico* analysis of all currently known HLA-I restricted minor H antigens, which have been identified using unbiased forward strategies, revealed that at least 28/48 of the non-immunogenic counterpart peptides are likely not expressed on the cell surface. This is probably an underestimation because various parameters that affect intracellular peptide processing were (largely) disregarded in this overview, such as proteosome-catalyzed peptide splicing, internal proteosomal cleavage, ERAP1 trimming (16, 83, 93). In addition, the prediction algorithms have been trained on positive datasets and therefore currently have limited power to predict the absence of processing. Taken together, this dataset suggests that more than 58% of genetically variable antigens are immunogenic because the non-immunogenic peptide is simply not present on the cell surface (Table 1). Mono-allelic (by some investigators called as “dominant”) presentation was also seen in mass spectrometry data of HLA-I derived peptides (77, 94).

Recently, SNP-induced differential processing was also found for an HLA-II restricted minor H antigen (95), indicating that development of HLA-II epitope processing prediction algorithms may be valuable for future identification of immunogenic HLA-II presented antigens.

Strikingly, in the exploding field of neoantigen prediction models, differential surface expression of mutated vs. wild type peptide is largely neglected, which might be a reason of the large number of false positive neoantigen predictions (Figure 1D). Thus, the addition of a specific differential surface presentation module, based on the molecular features of minor H antigens and their allelic counterparts, to the current prediction models may improve both minor H antigen and neoantigen reverse identification strategies.

## Conclusions and Future Directions

There is no doubt that the genetic alterations encoding hematopoietic minor H antigens in the allo-SCT setting and neoantigens in the autologous setting can induce potent T cell responses in patients. For clinical application of both antigen types, the most important challenge is to include sufficient patients in the clinical trials. It is currently unclear what the best strategy will be to provide the personalized immunotherapy necessary to target either of these antigens. Furthermore, there are also major challenges for their identification. The current T cell epitope prediction algorithms need significant improvement. We here postulate based on the published data of the last 25 years that also predictions for the non-immunogenic allelic or wild type peptide should be included in the algorithms. Selecting only those candidate peptides that will be differentially expressed at the cell surface may increase the success rate to detect or raise antigen-specific T cells from the naïve repertoire.

## Author Contributions

TM and RS developed the concept, designed, and wrote the manuscript. AX performed literature analyses and generated the figures.

### Conflict of Interest

The authors declare that the research was conducted in the absence of any commercial or financial relationships that could be construed as a potential conflict of interest.
